# Towards personalized trocar placement: assessment of patient-specific abdominal wall distension due to pneumoperitoneum

**DOI:** 10.1007/s11701-025-02757-9

**Published:** 2025-09-16

**Authors:** Sanne T. Gritter, Simon C. Baltus, Beerend G. A. Gerats, Can Ozan Tan, Jelmer M. Wolterink, Ivo A. M. J. Broeders

**Affiliations:** 1 Surgery Department, Meander Medical Centre, Maatweg, Amersfoort, 3818 TZ Utrecht The Netherlands; 2 Robotics and Mechatronics, University of Twente, Drienerlolaan, Enschede, 5722 NB Overijssel The Netherlands; 3 Department of Applied Mathematics, Technical Medicine Center, University of Twente, Drienerlolaan, Enschede, 5722 NB Overijssel The Netherlands

**Keywords:** Pneumoperitoneum, Minimally invasive surgery, Trocar, Preoperative planning

## Abstract

To quantify how pneumoperitoneum affects abdominal shape and how this is influenced by patient characteristics. Data from 73 patients undergoing minimally invasive (robot-assisted or laparoscopic) surgery were collected, including RGB-Depth (RGB-D) videos and patient characteristics (weight, height, sex, and age). Abdominal landmarks were ink-marked, and video analysis quantified global and regional wall distension. Global wall distension is defined as the mean displacement of all landmarks, and regional deformations are the mean displacements in specific regions, divided into medio-lateral and cranio-caudal regions. Associations between deformations and patient variables were evaluated using univariate and multivariate analyses. Pneumoperitoneum induced a mean global shape change of 3.6 cm (**± **0.9 cm), varying from 1.3 to 5.8 cm. Global wall distension correlated with height, sex, age, and medio-lateral region (*P*** < **0.05). Multivariate analysis identified height, age, and medio-lateral region as independent predictors. This study shows that abdominal wall distension due to pneumoperitoneum varies between patients, and depends on height, age, and abdominal region. This provides important insights into patient-specific abdominal wall distension due to pneumoperitoneum. These insights support personalized surgical planning and may inform patient-specific simulations for optimized trocar placement.

## Introduction

Minimally invasive procedures have a prominent role in today’s surgical practice. These techniques offer considerable advantages compared to open procedures, ultimately improving patient outcomes [1, 2]. In abdominal surgery, trocars are inserted through the abdominal wall to access the target anatomy. Accurate positioning of these trocars is critical for the successful execution of the procedure.

Trocar positioning affects both the freedom of movement and the distance to the target area. Consequently, inadequate trocar placement can lead to suboptimal surgical performance [3]. However, surgeons currently have limited support to achieve accurate trocar placement. While several studies have aimed to define guidelines to assist surgeons in optimal trocar placement, these guidelines are often based on generic patient images and do not account for abdominal wall distension caused by pneumoperitoneum [4–7]. Pneumoperitoneum is created by insufflating carbon dioxide into the peritoneal cavity, which causes distension of the abdominal wall, thus increasing the working space and visibility for the surgeon. In clinical practice, the trocars are positioned and applied through the abdominal wall *after* pneumoperitoneum, making it challenging to follow guidelines that are based on anatomy *before* insufflation.

Some studies have aimed to simulate the effects of pneumoperitoneum, but few have quantified the abdominal wall distension due to pneumoperitoneum. Song et al. studied abdominal wall distension to assess the mechanical properties of the abdominal wall, but this was done on a small dataset of 18 patients and no analysis of interpatient differences was included [8]. Quantifying abdominal wall distension is essential for understanding the effects of pneumoperitoneum, and eventually simulating this to create more interpretable, patient-specific guidance for trocar placement.

To address this gap, we created a multimodal dataset consisting of RGB-Depth (RGB-D) and electronic health record (EHR) data. This dataset enables the analysis of patient-specific abdominal wall distension due to pneumoperitoneum. Furthermore, we investigate how this relates to individual patient characteristics. The results of this study emphasize the need to develop a predictive model to support optimal, personalized trocar placement.

## Methods

### Data collection

Data were collected from patients who underwent minimally invasive diaphragmatic hernia repair or cholecystectomy surgery, robot-assisted or laparoscopic, and provided informed consent. A multimodal dataset was collected from each patient, involving an RGB-D recording of the pneumoperitoneum application and patient characteristics extracted from the EHR. The following characteristics were included: sex, age (years), height (cm), weight (kg), body mass index (BMI, kg/m^2^), and type of procedure.

#### RGB-D recordings

**Fig. 1:**
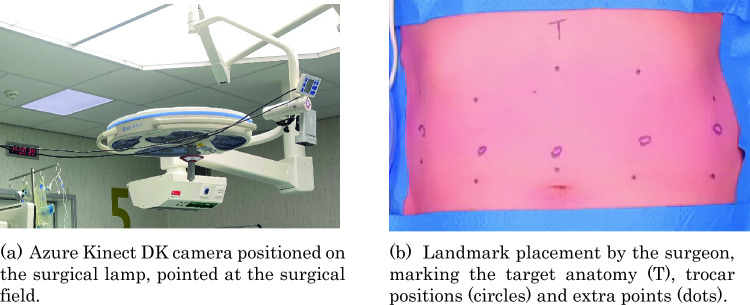
Overview of the data collection setup (**a**), and the ink landmarks placed by the surgeon (**b**)

The pneumoperitoneum application was recorded using an RGB-D camera (Microsoft Azure Kinect DK [9]), to capture the abdomen and its distension during this process. This camera records depth images using a time-of-flight sensor and combines them with RGB images to create a detailed three-dimensional (3D) RGB-D reconstruction over time. The camera was positioned on the surgical lamp using a clamp, capturing the surgical scene, from a distance of ± 1*.*5 m to the patient (Fig. 1a). All patients were recorded in a supine, horizontal position. Faces and other recognizable body parts were covered to ensure anonymity. The recordings were captured at five frames per second (fps), with a resolution of 1080 × 1080p and a depth mode set at near-field-of-view (FOV) unbinned.

After draping the sterile covers onto the patient, the surgeon placed ink landmarks on the abdomen, delineating the target anatomy (T), trocar positions (circles), and six-to-ten additional landmarks (dots), distributed across the visible quadrants of the abdomen to ensure spatial coverage (Fig. 1). The placement of the additional marks was not standardized but followed the principle of distributing landmarks evenly. Subsequently, pneumoperitoneum was established, and the surgical field was recorded until the insufflation pressure reached 15 mmHg.

### Data processing

**Fig. 2:**
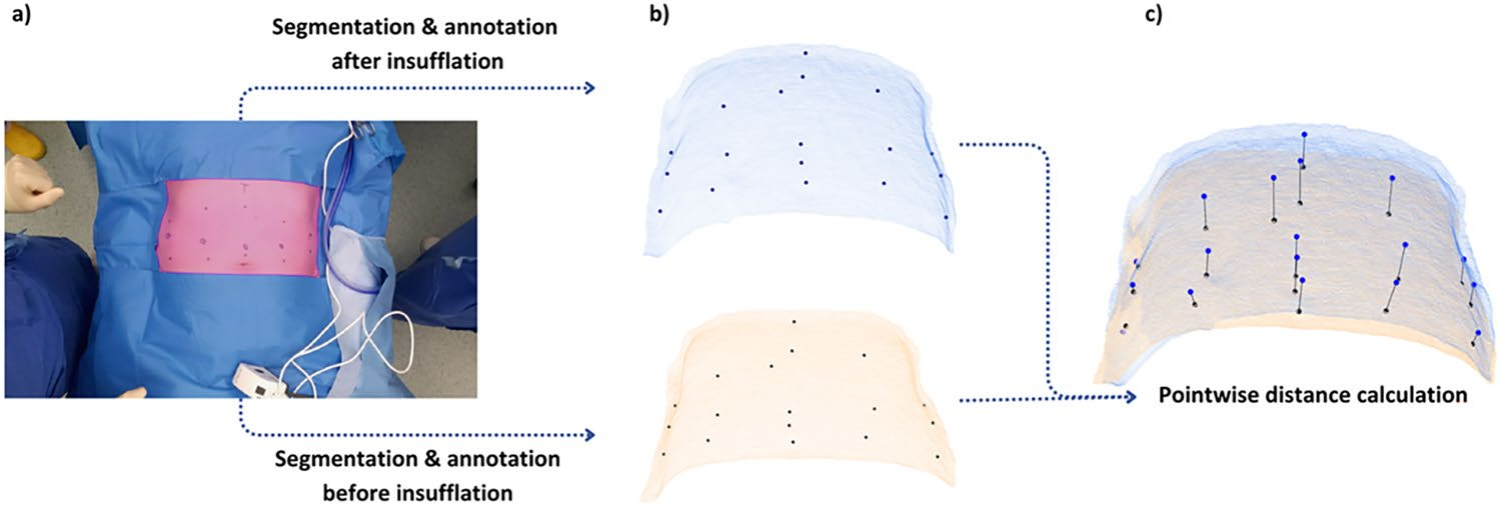
Workflow for quantification of the abdominal wall distension based on RGB-D recordings: **a** segmentation of the visible abdominal surface; **b** visualization of the segmented abdominal surface in 3D, including annotations of the ink landmarks on the abdominal surface; **c** overlay of the pre- and post-insufflation abdominal surfaces, where the line between the landmarks represents the Euclidean distance

We manually segmented the visible part of the abdomen in the RGB-D recordings, including an annotation of the positions of the ink landmarks, to create a 3D reconstruction of the abdomen. This segmentation and annotation process was executed for two time points: before and after insufflation. Pre- and post-insufflation time points were chosen without synchronization to the ventilatory cycle. The abdominal wall distension was quantified by computing the Euclidean distance of each landmark in the abdominal 3D reconstruction, from its pre-inflation to post-inflation position. This was done on a global level, taking the mean of all points per patient, and regional, defined as the mean of all points in a specific region. A visualization of this workflow is shown in Fig. 2.

**Fig. 3:**
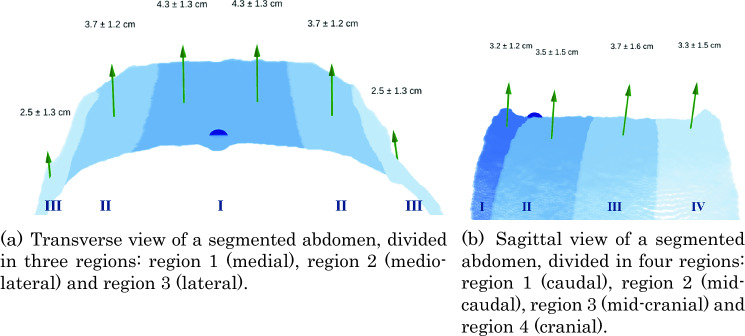
Visualizations of the mean abdominal wall distensions per region, with their directions. The mean distension vectors per region are visualized with green arrows, together with the means and standard deviations of the distension magnitudes. The dark blue dot represents the umbilicus

Regional abdominal wall distension was calculated by dividing the abdominal surface into predefined regions along two anatomical axes: medio-lateral and cranio-caudal. Medio-lateral regions are: central (region 1), mid-lateral (region 2), and lateral (region 3) (Fig. 3a). These regions are defined based on the symmetrical division between a midline drawn in cranio-caudal direction over the umbilicus, and the most lateral borders of the visible abdomen. Cranio-caudal regions are: caudal (region 1), mid-caudal (region 2), mid-cranial (region 3), and cranial (region 4). These regions are based on a division between a midline drawn in medio-lateral direction over the umbilicus. The surface above this line was divided into three regions (2, 3, 4), and the surface below this line was defined as region 1.

### Abdominal shape change analysis

#### Global abdominal wall distension

Normality of the data was assessed using the Kolmogorov–Smirnov test. Subsequently, the relationships between patient characteristics and global abdominal wall distension were evaluated using univariate and multivariate statistical analyses. Univariate analyses included Spearman’s rank correlation and Pearson correlation, depending on the data type and distribution. A multivariate analysis was performed using ordinary least-squares (OLS) regression to assess the combined effect of multiple patient characteristics. Associations were considered statistically significant at a P value of < 0.05. Variance inflation factor (VIF) was calculated to assess multicollinearity between predictors. A VIF lower than five was considered to indicate low collinearity.

#### Regional abdominal wall distension

To assess the differences in shape change across regions, the mean displacement of the landmarks within each region was compared using the Kruskal–Wallis test and Spearman’s rank correlation. Following the Kruskal–Wallis test, a Dunn’s post hoc test was executed to examine pairwise differences between all regions.

## Results

### Data collection

For this study, we collected data from 73 patients, of whom 14 underwent a laparoscopic cholecystectomy, 25 underwent laparoscopic diaphragmatic hernia repair, and 34 underwent a robotic diaphragmatic hernia repair. Eight patients were excluded due to camera movement or patient movement during the application of pneumoperitoneum, leaving 65 patients for final inclusion.

### Global and local displacement analysis

The RGB-D video analysis showed that the pneumoperitoneum led to a mean global abdominal wall distension of 3.6 cm (± 0*.*9 cm), with global distension per patient ranging from 1.3 to 5.8 cm. Figure 3 shows a visualization of the medio-lateral and cranio-caudal regions, with their mean distension magnitude and direction. In all regions, the distension is mainly in the anterior direction. In the medio-lateral direction, as visualized in Fig. 3a, central regions showed greater displacement (mean 4.2 cm), compared to lateral regions (mean 2.5 cm). The differences in displacement between the cranio-caudal regions were smaller (Fig. 3b).

### Correlation with patient characteristics

Table 1 presents the distribution of the patient characteristics, as well as their relationships with the global abdominal wall distension. Univariate analysis revealed significant correlations between global abdominal wall distension and patient height, sex, and age (*P* < 0.05). Male sex showed a negative correlation (*C* = − 0.258), whereas age (*C* = 0.247) and height (*C* = 0.312) correlated positively. Multivariate analysis showed that age and height are independent predictors for abdominal wall distension, with a VIF lower than five indicating no relevant multicollinearity.

**Table 1:** Univariate and multivariate correlation analyses of the relationship between the global abdominal wall distension and patient characteristics, as well as the abdominal regions, medio-lateral and cranio-caudal

Univariate and multivariate regression				
Variable	Overall (*n* = 65)	Univariate		Multivariate
		*P*	*C*	*P*
Sex (%)		0.040	− 0.258	> 0*.*05
Male	17 (26.2%)			
Female	48 (73.8%)			
Age (year)	61.6 (± 15*.*0)	0.049	0.247	< 0.001
Height (cm)	170.6 (± 8*.*5)	0.012	0.312	< 0.001
Weight (kg)	80.2(± 13*.*7)	> 0*.*05	0.102	> 0*.*05
BMI (kg/m^2^)	27.6 (± 4*.*5)	> 0*.*05	− 0.102	> 0*.*05

Regions	Kruskal–Wallis	Spearman’s *ρ*	Dunn’s post hoc	
	*H* (*P*)	*ρ* (*P*)	Individual regions	*P*
Medio-lateral	268 (< 0.001)	− 0.493 (< 0.001)		
			1 vs 2	< 0.001
			2 vs 3	< 0.001
			1 vs 3	< 0.001
Cranio-caudal	30 (< 0.001)	− 0.071 (0.022)		
			1 vs 2	0.004
			2 vs 3	0.037
			3 vs 4	0.032
			1 vs 3	> 0.05
			1 vs 4	> 0.05
			2 vs 4	< 0.001

The lower part of the table shows the results of the Kruskal–Wallis, Spearman’s *ρ*, and Dunn’s post hoc for the correlation between abdominal region and mean landmark displacement

*BMI* body mass index, *n* number of patients, *C* coefficient, *H* Kruskal–Wallis test statistic, ρ Spearman’s rank correlation coefficient

The Kruskal–Wallis test and Spearman’s rank correlation revealed significant differences in abdominal wall distension between regions, both in medio-lateral and in cranio-caudal direction. The differences are more pronounced in medio-lateral direction (*H*(2) = 268, *ρ* = − 0.493) than in cranio-caudal direction (*H*(3) = 30, *ρ* = − 0.071). The results of Dunn’s post hoc test revealed significant pairwise differences between nearly all regions, with the exception of cranio-caudal regions 1 vs 4 and 1 vs 3 (*P* > 0.05).

## Discussion

This study investigated abdominal wall distension due to pneumoperitoneum using RGB-D imaging. Abdominal wall distension varied considerably between individuals, with global distension ranging from 1.3 to 5.8 cm (mean 3.6 ± 0.9 cm). Distension occurred primarily in the anterior direction, with a greater magnitude in the central abdominal regions compared to the lateral regions. Univariate and multivariate analyses showed that global distension is independently associated with patient height, age, medio-lateral region, and cranio-caudal region, suggesting that both individual patient characteristics and location on the abdominal surface influence the abdominal wall distension due to pneumoperitoneum. The results of this study underscore the importance of patient-specific simulation of pneumoperitoneum to provide realistic, interpretable trocar placement guidance to surgeons.

The presented study shows that global wall distension is independently associated with patient height, age, medio-lateral region position, and cranio-caudal region position. This is in line with the findings of Malbrain et al., who described that abdominal compliance is higher in patients with increased height and age [10]. They also suggested that BMI and sex influence abdominal wall compliance, which contradicts the findings of the present study. Yildirim et al. also found a positive correlation between age and compliance, and no correlation between BMI and compliance, thus confirming our findings [11]. Furthermore, our observations that abdominal wall distension has greater magnitude in the medial regions are consistent with the previous reports [8, 12]. Those reports indicate that pneumoperitoneum mainly causes expansion in the central abdomen, likely due to higher compliance of the midline and uniform pressure distribution during pneumoperitoneum.

While port placement is performed based on a fully insufflated abdomen, our study findings offer insights for pre-operative insufflation simulation. This simulation can be used for education or personalized pre-operative surgical planning. The findings of this study offer two crucial implications. First, the measured variation in global wall distension between patients highlights the individual variations in abdominal wall distension due to pneumoperitoneum. These variations suggest that standardized pneumoperitoneum simulation may not be optimal for all patients, thus emphasizing the need for patient-specific pneumoperitoneum simulation methods. Second, we found that abdominal wall distension was independently associated with patient height and age. These relationships suggest that it may be possible to simulate abdominal wall distension preoperatively, using those patient characteristics. Additionally, we observed that lateral abdominal regions show smaller wall distension, compared to the medial part of the abdomen. This spatial variation in shape change can inform more precise surgical planning. Together, these insights contribute to a more personalized understanding of the operative scene and support the development of patient-specific simulations to guide trocar placement in minimally invasive surgery.

For patient-specific trocar placement, multiple factors must be considered, such as the procedure type, the modality (robotic vs. laparoscopic), the target organ, the patient’s abdominal shape, and how this shape changes due to abdominal wall distension caused by pneumoperitoneum. This study focused specifically on this abdominal wall distension due to pneumoperitoneum, and its relationship with patient characteristics. To our knowledge, this is the first study that used RGB-D cameras to generate realistic data to quantify the abdominal wall distension automatically and to assess its relationship with patient characteristics. Unlike previously used methods utilizing, for example, external motion tracking systems, our acquisition method enabled a non-invasive method of acquiring data without influencing patient treatment, surgery duration, or abdominal deformation [8].

As a next step, future research should aim to develop a patient-specific trocar placement planning approach that incorporates all the mentioned patient-specific factors. A first step in this planning would be the prediction of the abdominal shape, including its changes due to pneumoperitoneum, enabling the creation of an intuitive, realistic simulation of the intra-operative situation. Initially, this representation could assist surgeons during trocar placement. In the future, this knowledge could be used for automatic trocar placement planning. While recent studies have aimed to provide such a realistic simulation of the patient, including a simulation of pneumoperitoneum, most methods are based on mathematical models and/or validated on porcine data, instead of realistic human data [13–18]. The dataset collected in this study provides the opportunity to build and validate more accurate, human-based models.

### Limitations

This study has several limitations that warrant consideration. First, the quantification of abdominal shape change was based on a relatively small dataset, although this dataset is bigger than previous studies that investigated abdominal wall distension due to pneumoperitoneum [8, 12]. In our study population, BMI was not associated with abdominal wall distension. The current population had a relatively narrow BMI distribution (27.6 ± 4.5), which limits the assessments of the correlation. Assessment based on a more diverse group could prove a correlation between BMI and abdominal wall distension. Second, while patient characteristics extracted from the EHR were included in our analysis, prior research suggests that abdominal wall distension may also be influenced by factors observable in Computed Tomography (CT) imaging [12]. Future research should focus on incorporating a larger dataset with comprehensive CT imaging, to investigate the relationship between CT-derived features and abdominal wall distension due to pneumoperitoneum. Third, the visibility of the abdomen during application of pneumoperitoneum was limited. As the patients underwent upper abdominal procedures, the sterile drapes partially covered abdominal areas, specifically below the umbilicus and the lateral abdomen. The cranial edge of the sterile drapes was placed near the inferior border of the sternum. Mark placement and the subsequent analysis of regional wall distension were restricted to the visible surface, which led to the exclusion of a substantial portion of the abdomen. The limited field of view may also explain the lack of displacement in the lateral direction. In future studies, ensuring wider abdominal exposure or the inclusion of lower abdominal surgery could improve the accuracy and completeness of regional analysis. Finally, chest wall motion from mechanical ventilation may have contributed to minor additional movements of the abdominal wall. Although these movements were small compared to pneumoperitoneum-induced distension, we did not compensate for this effect.

## Conclusion

This study shows that abdominal wall distension due to pneumoperitoneum varies between patients, and depends on height, age, and abdominal region. This provides important insights into patient-specific abdominal wall distension due to pneumoperitoneum, highlighting the need for personalized surgical planning. Collectively, these insights contribute to a more personalized understanding of the operative scene and may support the development of patient-specific simulations of pneumoperitoneum, to eventually guide surgeons in optimal trocar placement.

## Data Availability

The dataset generated and analyzed during the current study is not publicly available due to privacy regulations and ethical restrictions related to patient data.
